# Electron Transfer in the Biogeochemical Sulfur Cycle

**DOI:** 10.3390/life14050591

**Published:** 2024-05-06

**Authors:** Xuliang Zhuang, Shijie Wang, Shanghua Wu

**Affiliations:** 1 Key Laboratory of Environmental Biotechnology, Research Center for Eco-Environmental Sciences, Chinese Academy of Sciences, Beijing 100085, China; xlzhuang@rcees.ac.cn (X.Z.); sjwang_st@rcees.ac.cn (S.W.); 2 College of Resources and Environment, University of Chinese Academy of Sciences, Beijing 100049, China; 3 State Key Laboratory of Tibetan Plateau Earth System, Environment and Resources (TPESER), Institute of Tibetan Plateau Research, Chinese Academy of Sciences, Beijing 100101, China

**Keywords:** biogeochemical sulfur cycle, electroactive microorganisms, electron transfer, long-distance electron transfer, cytochrome, pili

## Abstract

Microorganisms are key players in the global biogeochemical sulfur cycle. Among them, some have garnered particular attention due to their electrical activity and ability to perform extracellular electron transfer. A growing body of research has highlighted their extensive phylogenetic and metabolic diversity, revealing their crucial roles in ecological processes. In this review, we delve into the electron transfer process between sulfate-reducing bacteria and anaerobic alkane-oxidizing archaea, which facilitates growth within syntrophic communities. Furthermore, we review the phenomenon of long-distance electron transfer and potential extracellular electron transfer in multicellular filamentous sulfur-oxidizing bacteria. These bacteria, with their vast application prospects and ecological significance, play a pivotal role in various ecological processes. Subsequently, we discuss the important role of the pili/cytochrome for electron transfer and presented cutting-edge approaches for exploring and studying electroactive microorganisms. This review provides a comprehensive overview of electroactive microorganisms participating in the biogeochemical sulfur cycle. By examining their electron transfer mechanisms, and the potential ecological and applied implications, we offer novel insights into microbial sulfur metabolism, thereby advancing applications in the development of sustainable bioelectronics materials and bioremediation technologies.

## 1. Introduction

**Figure 1:**
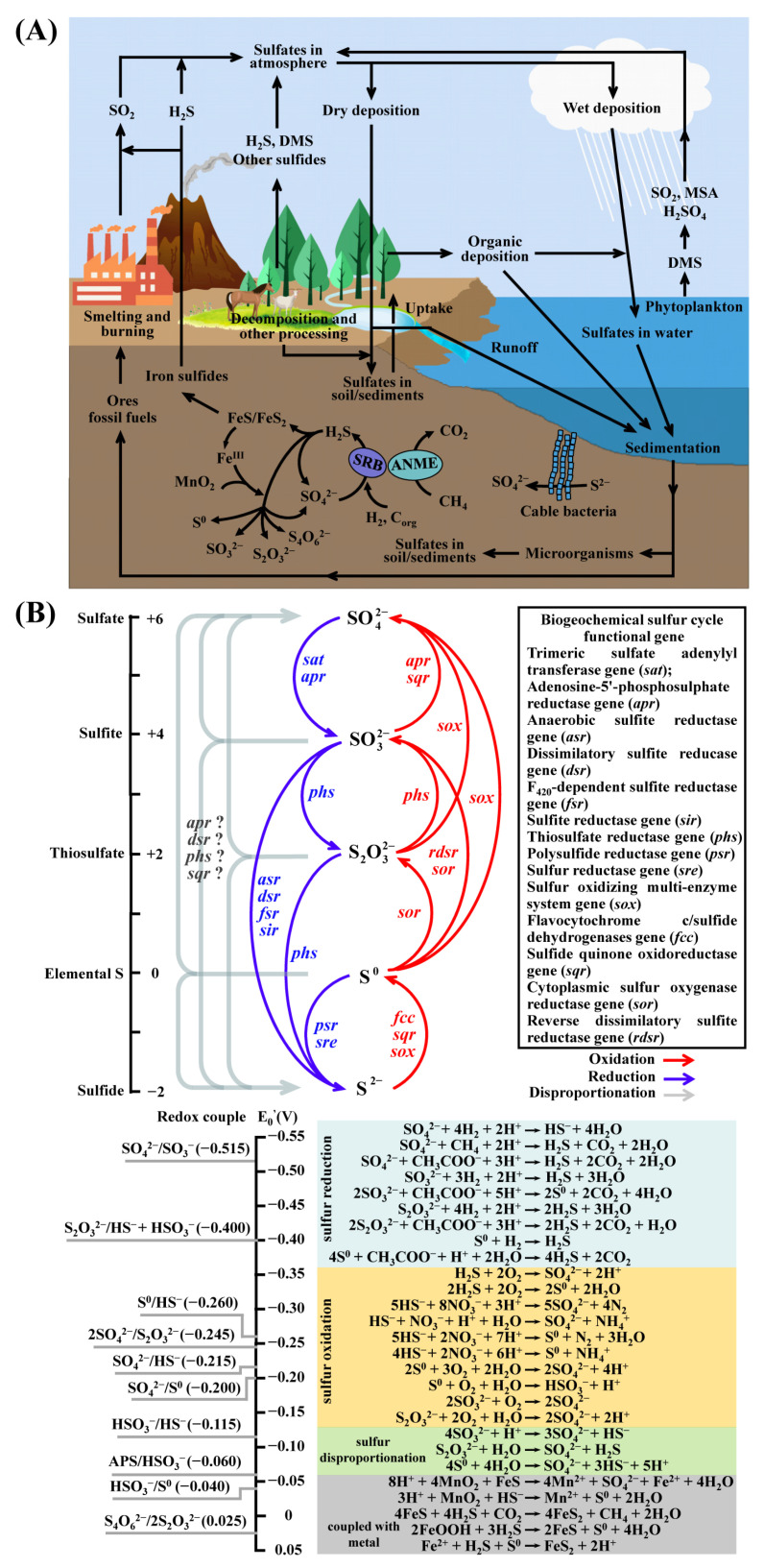
Conceptual diagram of the sulfur cycle. (**A**) Global sulfur cycle. The diagram includes some processes discussed in this review. Arrows indicate sulfur fluxes and pathways of biogeochemical or chemical processes. DMS, dimethyl sulfide; MSA, methyl sulfonic acid; C_org_, organic matter; ANME, anaerobic methane-oxidizing archaea; SRB, sulfate-reducing bacteria. (**B**) The biogeochemical cycle of key sulfur compounds. The schematic representation includes the microbially mediated reactions, half-reaction redox potentials [6,7,8], and functional genes involved in the biogeochemical sulfur cycle [5,9,10,11]. The question mark symbol means that the involvement gene is uncertain.

The sulfur cycle is a complex cycle with a fundamental role in the biogeochemical processes and redox reactions (Figure 1) [1]. The complexity is driven by different valence states of sulfur, ranging from −2 (sulfide and reduced organic sulfur) to +6 (sulfate). The existence of numerous redox states of sulfur enables countless transformations, and diverse microorganisms have evolved the capability to utilize these compounds within the biosphere (Figure 1B). Notably, dissimilatory metabolism, such as elemental sulfur reduction, sulfate reduction, sulfate disproportionation, and sulfide oxidation, not only fuels those microorganisms but also plays a crucial role in regulating the redox balance on the Earth’s surface [2,3,4,5].

The ability of microorganisms to perform extracellular electron transfer is a widespread natural process [12,13] and has been extensively studied in defined methanogenic co-cultures, including the co-cultures of *Geobacter metallireducens* and *Geobacter sulfurreducens* [14] and of *Methanosarcina barkeri* and *Geobacter metallireducens* [15]. Notably, this process is also widespread among microorganisms participating in the biogeochemical sulfur cycle, and it is currently attracting intense attention within a variety of research disciplines, such as bioelectrochemistry, microbial ecology, and cell physiology [16,17,18,19]. For instance, electron transfer occurs in consortia of sulfate-reducing bacteria and anaerobic methanotrophic archaea (ANMEs) [20,21,22], as well as in a novel multicellular filamentous bacterium known as cable bacteria [23]. These cable bacteria are not only capable of oxidizing sulfide through long-distance electron transfer (LDET) but can also receive electrons from other sulfur oxidizers [24,25]. These findings add to the complexity of the biogeochemical sulfur cycle while also explaining the key consequences of electron transfer in this cycle.

Since the initial discovery of electroactive microorganisms [26], more and more research has begun to pay attention to electron transfer mechanisms and mediated processes [27,28]. A few strategies have been proposed to mediate extracellular electron transfer in various microbial consortia. The best-known is mediated interspecies electron transfer (MIET), whereby soluble redox mediators (electron carriers) are released into the extracellular environment and reach other cells via diffusion along a concentration gradient [29]. MIET is a relatively slow and indirect process for electron transfer. It is similar to nutrient, carbon substrate, and information exchange between microorganisms. H_2_ and formate are generally regarded as the dominant electron carriers in MIET. An alternative to MIET is direct interspecies electron transfer (DIET). DIET-based electron transfer occurs through conductive pili or extracellular filaments known as bacterial nanowires [24], cell–cell connections [30], and outer surface c-type cytochromes [14]. Compared to MIET using H_2_ or formate to transfer electrons, DIET has various advantages, including more rapid electron transfer and the generation of more energy for syntrophic partners [31,32]. MIET and DIET are both important forms of extracellular electron transfer, prevalent across diverse environments (e.g., marine, wetland, and waste treatment facilities) [27,31,33,34], and play a significant role in numerous biogeochemical sulfur cycle processes [35,36,37,38].

The microbial strategies for extracellular electron transfer have evolved for billions of years [39]. Some studies now serve as inspiration not only for energy generation, such as in microbial electrolysis cells [40], but also for pollution control and ecological restoration, as exemplified by cable bacteria controlling greenhouse gas emissions [18]. Despite challenges in identifying novel electroactive microorganisms, sophisticated molecular analyses, combined with spectroscopy and microscopic characterization, are swiftly broadening our understanding of the diversity and environmental function of these microorganisms. In this review, we present new findings on microbial electron transfer within the biogeochemical sulfur cycle. We describe sulfur-transforming electroactive microorganisms, along with their physiological and environmental functions. Additionally, we elaborate on the mechanisms underlying these electron transfer processes. Furthermore, our review highlights the prevalence and significance of microbial electron transfer in environmental technologies and offers insights into potential application prospects.

## 2. Electron Transfer in Microbial Sulfate Reduction

Numerous sulfur transformations are enabled by the many redox states of sulfur. The sulfate-reducing activity that occurs for the incorporation of sulfide radicals into the biosynthetic cycle is known as assimilatory sulfate reduction. This reduction process is widespread among organisms and does not result in the production of sulfide. The sulfate-reducing activity that occurs during anaerobic respiration is known as dissimilatory sulfate reduction. This reduction process can function as synergistic agents in the sulfur cycle, leading to the formation of syntrophic associations. In dissimilatory sulfate reduction, sulfate serves as the terminal electron acceptor, ultimately resulting in the production of sulfide. Sulfate-reducing activity accounts for most organic carbon mineralization (about 80%) in the marine environment, and 97% of sulfide on Earth is produced by sulfate-reducing activity [41,42]. In addition to sulfate, dimethyl sulfoxide, which is one of the most abundant forms of methylated sulfur in marine systems, also serves as a viable electron acceptor during sulfur reduction [1,43].

**Figure 2:**
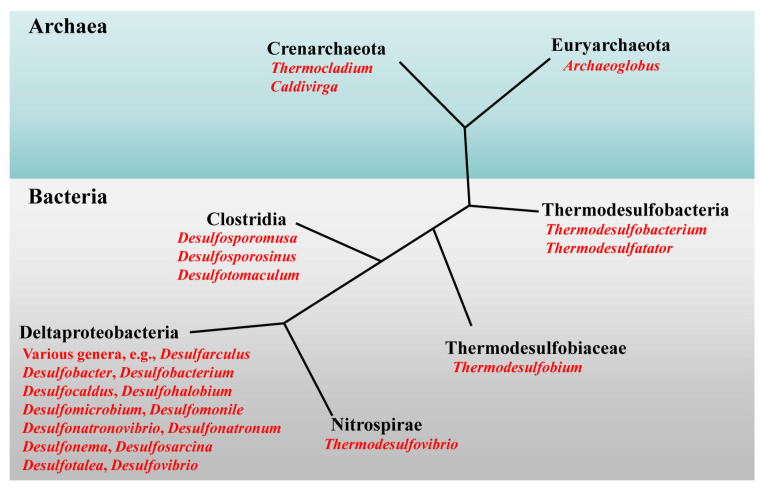
Schematic phylogenetic tree depicting the distribution of different types of sulfate-reducing microorganisms among major phylogenetic lineages. Note the seven phylogenetic lineages of sulfate-reducing bacteria, two in the archaea and five in the bacteria, and not all of the lineages with members capable of sulfate reduction are shown in the tree. The generic name is showed in red font.

Dissimilatory sulfur-reducing microorganisms have synergistic effects in the sulfur cycle and form syntrophic associations, for instance, growing syntrophically with methanogens [44]. Members of bacteria and archaea can use sulfate as a terminal electron acceptor. In this review, we use the term sulfate-reducing bacteria (SRBs) to refer to members of both domains. They are highly taxonomically diverse, and more than 220 species and 60 genera of SRBs have been recorded since 1895 [45,46,47]. SRBs can be found in many environmental locations including, but not confined to, seawater and marine sediments, wetlands, paddy soils, wastewater and many natural and engineered environments where sulfate is present [48,49]. Phylogenetically, SRBs can be assigned to seven lineages, including five bacterial and two archaeal lineages (Figure 2). Most SRBs belong to *Deltaproteobacteria*, including ~23 genera within the orders *Desulfovibrionales* and *Desulfobacterales*, followed by *Clostridia*, *Thermodesulfobacteria*, *Nitrospirae*, and *Thermodesulfobiaceae*. Within archaea, SRB belong to *Euryarchaeota* (genera *Archaeoglobus*) and to *Crenarchaeota* (genera *Thermocladium* and *Caldivirga*) [44]. In a recent study, the capacity for sulfate/sulfite reduction was identified in another 13 bacterial and archaeal phyla, expanding our understanding of the diversity of SRBs in the Earth’s ecosystems [50].

SRBs can use H_2_ and a number of organic compounds, such as formate, ethanol, lactate, pyruvate, succinate, and volatile fatty acids [51], as electron donors for sulfate reduction. Recently, a series of studies have shown that the anaerobic oxidation of methane (AOM) and other short-chain hydrocarbons can enable them to act as electron donors and can be coupled with sulfate reduction [16,52,53,54], and electron transfer plays an important role in this process. These findings highlight the role of interspecies electron transfer in the coupling of the biogeochemical sulfur cycle and organic carbon flux. AOM coupled with sulfate reduction was proposed in 1976 [55]. For decades, many studies have evaluated the microbiological mechanisms underlying this process. There is solid evidence that AOM is carried out by syntrophic consortia of archaea and SRBs. Thereinto, the processes of methane oxidation are carried out by anaerobic methane-oxidizing archaea (ANMEs): ANME-1; ANME-2a, b, c; and ANME-3 [33,56,57]. At the same time, these processes are coupled with sulfate reduction by a specific partner SRB [58,59,60], such as *Desulfosarcina*/*Desulfococcus* or members of the *Desulfobulbus* cluster. These results support an obligate functional role of the SRBs in AOM.

**Figure 3:**
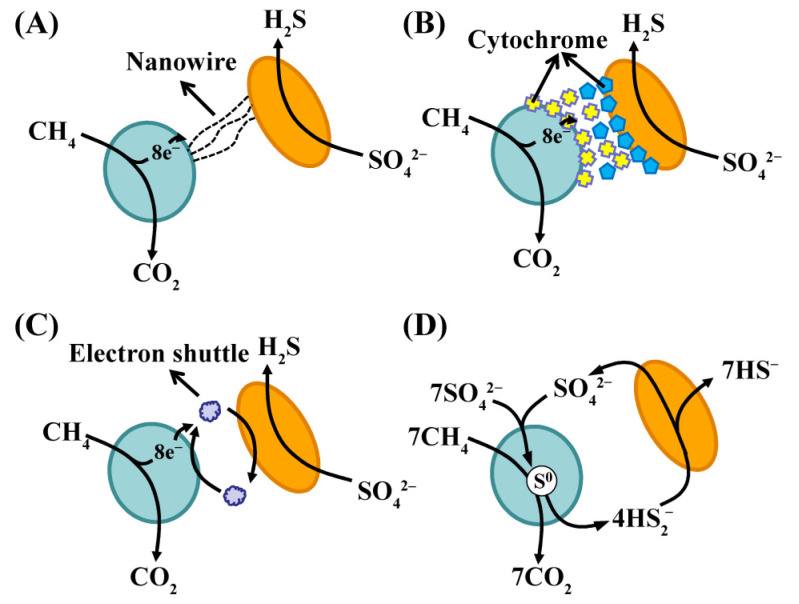
Mechanisms of intercellular electron transfer in consortia of ANMEs (blue) and syntrophic SRBs (orange). (**A**) Direct interspecies electron transfer via conductive nanowires. (**B**) Cytochrome-based direct electron transfer proposed for adjacent and/or non-adjacent cells. (**C**) Transfer of molecular electron shuttles. (**D**) Incomplete reduction of sulfate in ANMEs and zero-valent sulfur transfer to SRBs.

Previous studies have suggested a syntrophic coupling of ANMEs and SRBs via electron transfer [61]. ANMEs and SRBs always aggregate to form granule-like structures that probably facilitate electron exchange; however, the underlying mechanism for electron exchange remains unknown at this time. Initially, hydrogen was thought to be an electron carrier that facilitated MIET between taxa in different domains [62]. A recent investigation of a sediment-free thermophilic AOM enrichment consisting of ANME-1 and SRB HotSeep-1 has supported the notion that the process occurs via DIET [21]. Both ANME-1 and HotSeep-1 have genes encoding extracellular cytochrome production. Furthermore, the HotSeep-1 genome contains *pilA* [20], and these genes are highly expressed under thermophilic AOM conditions. A dense network of pili-like structures connecting HotSeep-1 to ANME-1 cells can be observed through transmission electron microscopy. The c-type cytochromes are predicted to interact with the extracellular S-layer of ANMEs [21]. A recent comparative genomics study also revealed that large multiheme cytochromes could be involved in DIET between ANMEs and SRBs [22]. Thus, the multi-heme c-type cytochromes and nanowires are likely responsible for electron transfer in this AOM consortium (Figure 3). Another study of the aggregates of ANME-2 and SRBs also provided evidence for syntrophic coupling through direct electron transfer [30]. This research speculated that these aggregates were electrically conductive because estimates of microbial activity fit a generalized model of electric conductivity between co-associated ANME-2 and SRBs. Metagenomic analysis and heme staining indicated the presence of large multi-heme cytochrome genes in the genomes of ANME-2 and c-type cytochromes not only in the membranes of ANME-2 and their SRB partners but also in the extracellular space between the cells. Based on this research, a proposed electron transfer model involves ANME-2 oxidizing methane and transferring electrons to extracellular cytochromes [63]. These extracellular cytochromes then establish a conductive matrix with outer surface cytochromes on the SRBs, enabling the SRBs to receive electrons and support sulfate reduction (Figure 3B).

However, electrically syntrophic coupling with SRBs might not be the only strategy for electron transfer by ANMEs. Previous studies have demonstrated that ANMEs can be decoupled from their syntrophic SRB partners using soluble artificial oxidants, such as anthraquinone-2,6-disulfonate (AQDS), humic acids, and Fe(III) complexes [63,64]. These studies also support the hypothesis that MIET occurs between ANME and SRB partners (Figure 3C). In addition to artificial oxidants, there is evidence that the intercellular sulfur cycle occurs between ANME-2 and SRBs during AOM, suggesting a new syntrophic mechanism [65] (Figure 3D).

Similar to methane oxidation from the association between ANME-1 and HotSeep-1 [30], the archaea *Candidatus* Syntrophoarchaeum can oxidize butane and possibly transfer electrons to SRB HotSeep-1 [54]. Using transmission electron microscopy, a dense, apparently pili-based, nanowire network was discovered in the intercellular space of the butane-oxidizing consortia. Additionally, in the enrichment culture, HotSeep-1 expressed genes encoding pili assembly proteins. Nanowire-based DIET (Figure 3A) was proposed for this butane-oxidizing consortium dominated by Ca. *Syntrophoarchaeum* and its electron-accepting partner HotSeep-1. HotSeep-1, also known as *Candidatus Desulfofervidus auxilii*, is a lithoautotrophic sulfate reducer [20]. Recently, a novel anaerobic ethane oxidizer, *Candidatus Ethanoperedens thermophilum*, and its syntrophic SRB partner *Candidatus Desulfofervidus auxilii* [16] were found in hydrothermal sediments. Based on genomic analysis, both microorganisms contain genes for cytochromes and pili, and they show a high expression of cytochromes and pili under ethane supply. It was proposed that the cytochromes and pili provide a structure for electron transfer in the syntrophic coupling of ethane oxidation with sulfate reduction. *Candidatus Argoarchaeum ethanivorans* is among the anaerobic ethane-oxidizing archaea, and it was obtained after specific enrichment from marine hydrocarbon seeps [53]. The sulfate-reducing *Deltaproteobacteria* were the only partners detected in the enrichment. However, direct electron transfer is not the potential syntrophic mechanism of the co-culture. Instead, the high enrichment of sulfur in Ca. *Argoarchaeum* cells suggests that an intercellular sulfur cycle similar to that found in consortia of ANME-2 and SRBs (Figure 3D) could be the syntrophic mechanism.

As discussed above, numerous studies demonstrating the role of SRBs in electroactive syntrophic consortia have expanded the diversity of known microbial sulfur transformations and highlight the importance of electron transfer in the coupling of biogeochemical sulfur and carbon cycles.

## 3. Electron Transfer in Microbial Sulfur Oxidation

Sulfur compounds such as elemental sulfur, sulfide, and thiosulfate can be utilized as energy sources by sulfur-oxidizing bacterial (SOB) groups. Broadly, these groups can be classified into two major categories: colorless and colored ones [66,67]. The colorless sulfur bacteria are a highly diverse, heterogeneous group [68], and they are widely present in various environments, such as marine, paddy, soil, and mine drainage [67,69,70]. They lack photopigments and can be categorized into obligate chemolithotrophs, facultative chemolithotrophs, and chemolithoheterotrophs [71]. Colorless SOB play an essential role in sulfur oxidation. Certain species of SOB are able to utilize oxidized forms of nitrogen, such as nitrates or nitrites, as electron acceptors, thus being considered autotrophic and denitrifying. This capability renders them extensively applied in environmental engineering for the removal of sulfide and nitrate from various water environments [67,72]. The colored SOB can be classified into green sulfur bacteria and purple sulfur bacteria. These SOB thrive in anoxic marine and freshwater environments characterized by both sulfide and light [73]. They are important in the sulfur cycle and as primary producers in environments with high sulfur concentrations, ones which can oxidize sulfide, thiosulfate, and elemental sulfur for photosynthetic growth (anoxygenic photosynthetic CO_2_ fixation) [74]. Green sulfur bacteria contain dissimilatory sulfite reductase genes and are capable of oxidizing elemental sulfur, thiosulfate, and sulfide [75,76]. Purple sulfur bacteria can also use organic compounds, making them facultative photolithotrophs [67].

**Figure 4:**
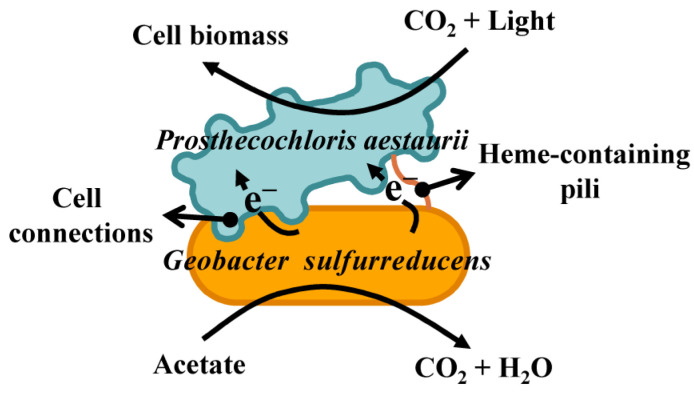
Mechanisms for syntrophic anaerobic photosynthesis of the green sulfur bacteria *Prosthecochloris aestaurii* and *Geobacter sulfurreducens* via direct interspecies electron transfer.

For SOB, electron transfer can offer an alternative strategy for growth. A recent study of syntrophic anaerobic photosynthesis has shown that the phototrophic green sulfur bacterium *Prosthecochloris aestuarii* can accept electrons from acetate oxidation via DIET from *Geobacter sulfurreducens* or from a solid electrode [77]. The co-culture showed intimate cell-to-cell connection and abundant heme-stained filamentous structures connecting *P. aestuarii* and *G. sulfurreducens*. Meanwhile, *P*. aestuarii does not grow in co-culture with a *G*. sulfurreducens deletion mutant lacking a trans-outer membrane porin-cytochrome protein complex required for DIET. This suggests that heme-containing proteins support DIET between cells and provide a mechanism for syntrophic anaerobic photosynthesis (Figure 4) [77]. In co-culture, *G. sulfurreducens* is the most important electrogenic bacterium, with the ability to transfer electrons directly to extracellular electrodes [14,78]. In addition, *G. sulfurreducens* is capable of reducing elemental sulfur [79]. Syntrophic anaerobic photosynthesis broadens the metabolic capacity of green sulfur bacteria and expands the concept of photosynthesis to include electron transfer between phototrophs and heterotrophs. Given that green and purple bacteria are widely distributed in anoxic environments [73], other taxa may also establish syntrophy with various electrogenic organisms via DIET.

**Figure 5:**
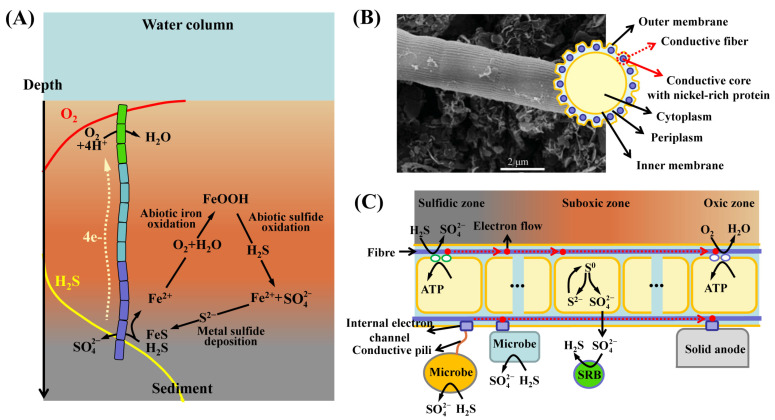
Unique metabolic characteristics of cable bacteria, and mechanism of electron transfer through cable bacterium filaments. (**A**) Model of electrogenic sulfur oxidation by cable bacteria in sediment, and a schematic view of effects of the cable on sedimentary iron and sulfur cycling. Long-distance electron transfer allows anodic cells in the anoxic zone to oxidize sulfide, and electrons transferred through the cable bacteria to cathodic cells extend into the oxic zone, where they reduce oxygen or nitrate. (**B**) Scanning electron microscopy images of the cable bacterium filaments show parallel ridges [83]. Schematic of the structure of a cross-section, revealing that the periplasm of cable bacteria contains a network of conductive fibers. (**C**) Model of long-range electron transfer inside the cable bacterium filament and proposed energy metabolism. Putative interactions between partner bacterium/solid anode and cable bacteria through direct contact or conductive pili.

In addition to the SOB mentioned above, a novel multicellular filamentous bacterium called cable bacteria was discovered, which can oxidize sulfide via LDET [23,80]. Cable bacteria are found worldwide in marine and freshwater sediments [81,82,83,84,85,86]. Based on a 16S rRNA gene sequence phylogeny, cable bacteria belong to the deltaproteobacterial family *Desulfobulbaceae*, including the two genera *Candidatus* Electrothrix and *Candidatus* Electronema and nine described candidate species [23,87]. Cable bacteria span the vertical gap between sulfide and oxygen; the main part oxidizes sulfide in the subsurface layer, and resulting electrons are transferred to the top of the cells through filaments, which transfer the electrons to oxygen or nitrate near the uppermost sediments (Figure 5A). Therefore, cable bacteria separate the redox process of sulfide oxidation into two reactions and oxidize sulfide without immediate access to the oxidant [88,89].

The occurrence of LDET within cable bacteria is supported by various observations. Cable bacteria contain a network of parallel periplasmic fibers [90], and these are continuous across cell-to-cell junctions (Figure 5B). Because these continuous periplasmic fibers run across the whole filament, they are prime candidates for electron transfer in cable bacteria. Recently, a series of studies have shown that electron transfer occurs via highly conductive fibers (Figure 5B,C) [17,80,90,91]. Bjerg et al. [91] used resonance Raman microscopy to analyze cytochrome redox states in living cable bacteria and found that the gradients in cytochrome redox states depended on an intact electrical connection between the electron donor H_2_S and the electron acceptor O_2_. Meysman et al. discovered high conductivity of periplasmic fibers through direct electrical and electrochemical measurements of intact filaments [17]. Combining high-resolution microscopy, spectroscopy, and chemical imaging indicated that the periplasmic fibers consist of a conductive protein core containing a sulfur-ligated nickel group (Figure 5B). The LDET in cable bacteria is crucially dependent on these proteins [80]. Recently, Digel et al. extracted the fibers from cable bacteria and used them as free-standing biobased electrodes [19]. They observed that these fibers can catalyze the reversible interconversion of oxygen and water through electron transfer. All these studies provide direct evidence for LDET in cable bacteria. Given that cable bacteria are up to one centimeter long, electron transfer largely exceeds the maximal distance observed, i.e., micrometer-scale distances in *Geobacter* [92], and suggests that biological evolution has resulted in an organic structure that is capable of highly efficient electron transfer across centimeter distances.

Cable bacteria may also participate in DIET. A previous study has suggested that chemolithoautotrophic *Epsilon*- and *Gammaproteobacteria* can oxidize sulfur when cable bacteria are present [24]. These sulfur oxidizers may transfer electrons to the cable bacteria via DIET (Figure 5C); however, the electrical connection needs to be further determined. A previous study of benthic microbial fuel cells has shown that cable bacteria can attach to the solid anode (Figure 5C), which serves as an electron acceptor [93]. In benthic microbial fuel cells, cable bacteria may interact with other electrogenic bacteria (e.g., in the family *Desulfuromonadaceae*) through extracellular electron transfer. Recently, Bjerg et al. discovered through Raman microscopy that diverse bacteria form a tightly packed flock around the anoxic part of cable bacteria in a freshwater sediment enrichment culture [25]. Further analysis indicated that these flocking bacteria included sulfide oxidizers, which might transfer electrons to cable bacteria for sulfur oxidation. In some instances, sulfur disproportionation (Figure 5C) may largely represent the energy metabolism of cable bacterial filaments [94].

However, despite enrichment efforts, cable bacterial species have not been isolated in pure culture [87]. Accordingly, the specific contribution and key role of cable bacteria to microbial communities associated with electron transfer in aquatic sediments is not well-understood. Therefore, the isolation of cable bacteria remains a key focus of research to gain a deeper understanding of their growth modes and ecological significance. Furthermore, the identification of culture strategies has the potential to contribute to the development of microbial fuel cells.

## 4. Role of Pili/Cytochrome in Electron Transfer

The ability of microorganisms to express conductive pili or similar nanowires is an effective predictor of electron transfer in electroactive communities (e-communities). *Geobacter metallireducens* and *G. sulfurreducens* express conductive pili as the electron transfer structure for DIET. In the consortia of ANME-1 and SRB HotSeep-1, a nanowire-like structure was proposed to transfer electrons from ANMEs [20]. Electrically conductive nanowires were also observed in the iron-respiring bacterium *Rhodopseudomonas palustris* strain RP2 [95]. Thus, the pili/nanowire structure likely has a significant role in electron transfer. However, some *G. metallireducens* and *G. uraniireducens* strains could not be grown via DIET [96,97], likely because the pili are poorly conductive. Previous structural analyses have revealed that, during extracellular electron transfer, *Geobacter* species cells produce nanowires comprising the cytochromes OmcS and OmcZ [98,99]. Additionally, they produce heterodimeric pili, but these pili exhibit a conductivity that is 20,000-fold lower compared to that of OmcZ nanowires [100]. These findings make the necessity of pili or nanowires in electron transfer ambiguous, as different structures of pili/nanowires can lead to significant variability in their conductivity, which further extends their effects within microbial aggregates. Despite extensive studies of the e-pili of *Geobacter* species and the nanowires of HotSeep-1, these recent evolutionary events are not representative of the wide diversity of microorganisms capable of electrical communication in syntrophic consortia [20,101].

Cytochromes are multi-heme-binding cell-bound proteins that play an important role in intracellular electron transport and oxidation. The important role of cytochromes in electron transfer is clear; for example, multicellular consortia of ANME-2 with SRB partners show regions of dense heme-staining, which indicates cytochrome-based electron transfer between the two domains [30]. Furthermore, ANME genomes contain many more genes encoding multi-heme cytochromes than any of their methanogenic relatives [22,102]; this indicates that cytochromes may perform an electron transfer function in the extracellular space between ANMEs and SRBs, which is consistent with the above study. The outer membrane cytochrome OmcB of *G. sulfurreducens* is necessary for syntrophic anaerobic photosynthesis [77]. The aggregation of pili-free *Geobacter* species via DIET is mediated by Gmet_2896 cytochrome [103]. In rice paddy soils [104], methanogenic digestion [34], and anaerobic bioreactors for brewery wastewater [105], some e-communities contain abundant *Geobacter*, characterized by abundant c-type cytochromes. In addition, SRBs may exhibit direct extracellular electron uptake from solid electron donors via outer-membrane cytochromes [106]. The cytochromes involved in DIET are widely distributed. For example, cytochromes are the key means of extracellular electron transfer in many metal-reducing bacteria [107,108]. However, conductive pili or similar nanowires are restricted to some special microorganisms [109]. Most pili that extend from the cell surface exhibit the ability to assist with cell adhesion to surfaces, facilitate twitching motility, and transfer DNA between cells via conjugation [110]. Thus, the cytochromes perhaps have a more important role in electron transfer. The abundance of cytochromes in the extracellular matrix may also serve as a criterion for identifying e-communities via electron transfer.

## 5. Significance of Microbial Electron Transfer in the Environment and Applications

The biogeochemical sulfur cycle is highly intricate, involving a vast array of microorganisms, and this key cycle is further facilitated by microbial electron transfer. In the marine environment, SRBs are believed to play critical roles in coupled biogeochemical element cycles by biotic or abiotic processes [111]. For instance, the metabolic activity of SRBs is intimately linked to the process of hydrocarbon oxidation. Massive amounts of natural gas migrate from deep marine sediment towards the seafloor [112]; however, most of this gas is consumed in the anoxic zone by microorganisms coupling the oxidation of hydrocarbons and reduction of sulfate. Research on the anaerobic oxidation of natural gas has focused largely on methane and AOM [33,61,113,114]. AOM in marine environments is mainly sulfate-dependent [58]. ANMEs oxidize methane to CO_2_ by reversing the enzymatic chain of methanogenesis [36], while electrons are transferred to the partner SRBs by various mechanisms, such as MIET and DIET. AOM plays an important role in controlling methane emissions, consuming more than 90% methane produced from the seafloor, making the ocean a minor methane source (<2% of the global flux) [58,112], and 50% of methane from freshwater wetlands [115]. Indeed, it has been estimated that AOM supports 3% to 40% of sulfate reduction in marine sediments [116], suggesting that electron transfer plays a critical role in coupling the biogeochemical cycles of sulfur and carbon.

In addition to methane, ethane and butane are important natural gas components generated by the thermogenic decomposition of organic matter [117]. Both *Candidatus* Ethanoperedens thermophilum and Ca. Syntrophoarchaeum express genes encoding methyl-coenzyme M reductase [16,54], allowing for the oxidation of ethane and butane, respectively. Electrons seem to be transferred to partner SRBs, which use these to reduce sulfate. Genes encoding 16S rRNA and methyl-coenzyme M reductase, similar to those of Ca. Syntrophoarchaeum, have been repeatedly retrieved from marine subsurface sediments. Specifically, 16S rRNA gene sequences clustering with Ca. Ethanoperedens and Ca. Syntrophoarchaeum have been repeatedly retrieved from subsurface marine sediments in cold-seep and hot-vent environments [16,54], suggesting that interspecies electron transfer is naturally widespread in the anaerobic oxidation of these short-chain hydrocarbons.

The discovery of syntrophic anaerobic photosynthesis vastly expands the potential roles of DIET in nature and broadens the concept of electron transfer. Typically, studies of syntrophic interspecies electron transfer have focused on heterotrophic carbon metabolism [27]. Syntrophic anaerobic photosynthesis, which directly links anaerobic photosynthesis to anaerobic heterotrophic carbon metabolism, reveals a novel form of syntrophy. From an ecological perspective, syntrophic anaerobic photosynthesis could become an alternative metabolic process for phototrophs and heterotrophs, when limited sulfide and inorganic electron acceptors restrict the activity of anoxygenic phototrophs and anaerobic respiration. From the perspective of practical applications, this discovery is promising for bioenergy production and waste treatment.

The activity of cable bacteria (LDET) can increase the availability of sulfate and provides a strategy for recycling this scarce resource [84,118]. In addition to the sulfur cycle, cable bacteria can impact the coupling of sedimentary biogeochemical sulfur and iron cycles, thereby delaying the onset of euxinia in coastal waters [119]. A recent study revealed that cable bacterial metabolism reinforced their associated interspecific interactions with functional microorganisms such as sulfate reducers, polycyclic aromatic hydrocarbon degraders, and electroactive microbes, suggesting enhanced microbial syntrophy taking advantage of LDET [118]. Moreover, the inoculation of rice paddy soil with cable bacteria reduced methane emissions by increasing the sulfate inventory and stimulating sulfate reduction; SRBs, therefore, had a competitive advantage over methanogens for common substrates [18]. Rice fields release huge amounts of methane, accounting for approximately 11% of the global anthropogenic methane. Promoting cable bacteria in rice fields may thus become an economically and environmentally sound approach for mitigating greenhouse gas emissions [18]. Meanwhile, a recent study has discovered that cable bacteria mediating electrogenic sulfur oxidation can enhance the bioavailability of pyrene and promote the enrichment of degradative bacteria, thereby facilitating the removal of pyrene [82]. Therefore, the unique biological characteristics of cable bacteria endow them with a significant role in global biogeochemical cycles, and the LDET is advantageous for a variety of applications, such as microbial fuel cells for electricity generation or bioremediation of organic contaminants in sediments [25].

As discussed above, microbial extracellular electron transfer is important for the coupling of the sulfur cycle and other biogeochemical cycles. These processes not only help control the emission of greenhouse gases, such as methane, ethane, and butane, but also contribute to maintaining ecosystem functions. Just as the rapid transportation of electrons through a power grid has significantly improved the quality of human life, there has been a growing interest in electroactive microorganisms due to their potential applications in green technologies, particularly those dealing with renewable energy and environmental management [39]. In terms of applications, the ability of electroactive microorganisms to directly transfer electrons has been exploited over the last decade in bioelectrochemical systems. These systems encompass various technologies, such as microbial fuel cells, microbial electrolysis cells, and microbial electrosynthesis [40]. They are capable of reducing pollutants, facilitating bioremediation, recycling elements, synthesizing new products, and generating electricity [120,121,122,123,124]. For example, electroactive microorganisms release electrons capable of oxidizing and transforming organic matter and contaminants present in organic carbon-rich water, wastewater, soil, or sediment [125]. The use of microbial fuel cells has shown promise as a sustainable technology for simultaneous energy generation and wastewater treatment [126,127,128]. Electrically conductive pili, harvested from cells as ‘protein nanowires’, have potential as a novel electronic material [129,130]. Protein nanowires possess numerous advantages compared to traditional silicon nanowires or carbon nanotubes, such as greater flexibility in tuning conductivity and sustainable production from renewable feedstocks, resulting in a final product containing no toxic components [39]. Given that cable bacteria assemble the most impressive longer conductive filaments, they are definitely a promising form of functional bacterium for nanowire synthesis.

However, most of these technologies are still in the early stages and face substantial challenges before they can be approved for commercial application. Thus, identifying more extensive electroactive microorganisms and DIET mechanisms may help lead to improvements in practical applications [131].

## 6. Conclusions and Perspectives

In this review, we have summarized the current research on electron transfer in the biogeochemical sulfur cycle, for instance, the DIET between SRBs and anaerobic alkane oxidizing archaea, and the LDET and DIET in cable bacteria. Nevertheless, research related to electron transfer in the biogeochemical sulfur cycle remains limited, especially in in situ environments. It is estimated that there are thousands of electroactive microorganisms hiding in diverse ecosystems that needed to be explored [132]. The identification of new microorganisms capable of electron transfer and related e-communities should be a key focus of future research. As discussed above, significant improvements have been made in measuring the conductivity of pili or nanowires. However, when dealing with microbial aggregates or in situ environments that possess complex physical and chemical conditions, accurately measuring their conductivity, as well as identifying the underlying electroactive microorganisms, becomes more challenging. The novel LDET in individual cable bacteria [91] demonstrates that the pathway of electron transfer and the involved sulfur cycle processes exhibit significant diversity, meriting further study. Additionally, the extensive taxonomic diversity makes it difficult to develop RNA-based biomarkers for the identification of electroactive microorganisms, such as the conserved functional *Dsr* gene of SRBs. Consequently, there remain obstacles in identifying novel microorganisms capable of electron transfer, particularly due to the absence of precise screening methodologies.

However, as more is discovered about the microorganisms capable of electron transfer, gene expression patterns in e-communities, and mechanisms underlying electron transfer, we may be able to detect microbial electron transfer more extensively through advanced sequencing technologies or meta-omic approaches. For example, electric characterization, spectroscopy characterization, and microscopic characterization are potentially useful tools for exploring electroactive microorganisms and the detailed mechanism of DIET [17,133]. Microbial extracellular electron transfer greatly relies on the structure and electrochemical properties of redox proteins or shuttles, such as cytochromes, which are involved in nanowires and aggregates. Spectroscopic methods enable the analysis of the redox state of cytochromes or the identification of specific cytochromes [134,135]. Microscopy is also a valuable tool for visualizing nanowires and their activity [136]. Additionally, electrochemical measurement methods are crucial for characterizing the electrochemical activity of redox proteins or nanowires [137]. By combining recent molecular microbiological approaches, such as genome editing or sequencing [100,138], isotope probing coupled with Raman-activated cell sorting [139,140], and microbiomics integrated with data mining [141,142], we can develop a powerful tool for identifying key proteins or extracellular components expressed by microorganisms involved in electron transfer. Additionally, this combined approach can facilitate the faster and more accurate discovery of novel electroactive microorganisms. Meanwhile, to directly demonstrate the DIET between the electroactive microorganisms, isolates of the microbes and mutant strains (e.g., cytochrome and/or nanowire deletion mutants) and electrical measurements are still required.

In conclusion, microorganisms play a crucial role in the oxidative and reductive cycles of sulfur. Both intracellular and extracellular electron transfers are significant in the sulfur cycle and offer promising approaches for treating pollution and producing bioenergy. Studies of electroactive microorganisms are in an early stage. Recent advances in microoptic, bioinformatic, and omic techniques can improve our understanding of the biological, physiological, and biochemical properties of microbial electron transfer.

## Data Availability

Not applicable.
